# Effect of Warm Acupuncture Combined with Bone Marrow Mesenchymal Stem Cells Transplantation on Cartilage Tissue in Rabbit Knee Osteoarthritis

**DOI:** 10.1155/2021/5523726

**Published:** 2021-08-11

**Authors:** Jun-wei Liu, Yong-li Wu, Wei Wei, Yan-ling Zhang, Di Liu, Xiao-xiu Ma, Chun Li, Yu-yuan Ma

**Affiliations:** ^1^Department of Traditional Chinese Medicine Orthopedics and Traumatology, General Hospital of Ningxia Medical University, Yinchuan, Ningxia Hui Autonomous Region 750004, China; ^2^Key Laboratory of Hui Ethnic Medicine Modernization, Ministry of Education, Ningxia Medical University, Yinchuan, Ningxia Hui Autonomous Region 750004, China; ^3^Ningxia Medical University, Yinchuan, Ningxia Hui Autonomous Region 750004, China

## Abstract

The current study was designed to investigate the effect and underlying mechanism of warm acupuncture combined with bone marrow mesenchymal stem cells (BMSC) transplantation on cartilage tissue injury in rabbit knee osteoarthritis (KOA). In the study, 50 rabbits were randomly divided into 5 groups: blank group, KOA group, warm acupuncture group, BMSCs group, and warm acupuncture combined with BMSCs group. After warm acupuncture combined with BMSCs, the Modified Lequesne MG knee joint assessment scale was used to evaluate the degree of knee joint behavior, the Taiping Peng method generally observed the histomorphology changes of KOA rabbit cartilage, and hematoxylin-eosin staining, safranin O green staining, and toluidine blue staining were conducted to evaluate the extent of cartilage tissue pathology. Furthermore, transmission electron microscopy and TUNEL staining were used to observe cell apoptosis, and immunohistochemistry and qPCR analysis were used to detect the expression of apoptosis-related proteins and mRNA. Results showed that administration of warm acupuncture combined with BMSCs recovered the joint function and significantly decreased Lequesne MG score. The degree of cartilage tissue pathological damage has been improved, cartilage ultrastructure degeneration has recovered, peripheral blood vessels have mild edema, blood supply has gradually recovered, and even small amounts of red blood cells have appeared. In addition, warm acupuncture combined with BMSCs treatment suppressed chondrocyte apoptosis in rabbits with knee osteoarthritis by reduced TUNEL-positive chondrocytes and simultaneously reversed the mRNA expression of Bax, Bcl-2, and Caspase-3. These results indicate that warm acupuncture combined with BMSCs transplantation has a potential protective effect on rabbit KOA, which may be mediated by inhibiting chondrocyte apoptosis.

## 1. Introduction

Knee osteoarthritis (KOA) is a disease that causes articular cartilage tissue damage and joint dysfunction due to abnormal apoptosis of the chondrocyte [1]. With the advent of an aging society, the incidence of KOA shows an upward trend year by year [2, 3]. Therefore, there is an urgent need to formulate effective intervention strategies to prevent and reverse KOA, promote repair, and improve prognosis.

The articular cartilage regeneration ability of the knee joint is weak and cannot be repaired by itself after pathological changes occur. If not treated in time, the degree of articular cartilage damage will gradually worsen, and joint function may even be lost. Excessive apoptosis of chondrocytes is an important reason for the degeneration of articular cartilage into osteoarthritis [4–6]. Under physiological conditions, the proliferation and apoptosis of articular cartilage cells are relatively balanced, which can maintain the stability of the articular cartilage environment. When cell apoptosis is greater than proliferation, it will accelerate the degeneration of articular cartilage [7–9]. Therefore, antiapoptotic therapies via inhibiting excessive apoptosis of chondrocyte and maintaining the balance between chondrocyte proliferation and apoptosis have broad application prospects in reducing KOA.

Bone marrow mesenchymal stem cells (BMSCs) have the characteristics of stromal cells and secrete a variety of cytokines [10–12]. The secreted cytokines can inhibit local inflammation and improve the microenvironment of the injured site. In addition, BMSCs can repair injured sites by differentiating into corresponding cells [13]. In recent years, BMSCs with multidifferentiation potential have been used to induce differentiation into chondrocytes, participate in cartilage tissue reconstruction and repair, and inhibit chondrocyte apoptosis, which is of great significance in the treatment of KOA cartilage injury [14, 15]. The differentiation of BMSCs into chondrocytes is a complex process, which is affected by the concentration of cytokines and the microenvironment of the injured site [16]. The pathological environment of degenerative osteoarthritis is hypoxia and acidity, which contains a large number of inflammatory mediators, such as interleukin and nitric oxide. Poor intra-articular microenvironment endangers the viability of BMSCs after transplantation, resulting in BMSCs being vulnerable to injury, apoptosis, or necrosis after implantation, thereby reducing the viability of transplantation [17, 18]. Therefore, seeking a way to improve the microenvironment of KOA joints, inhibit chondrocyte apoptosis, and alleviate KOA is an urgent problem to be solved.

In the past few decades, traditional Chinese medicine has been widely used in the clinical treatment of KOA due to its diverse treatment methods, positive curative effects, few side effects, and low cost. Warm acupuncture as a commonly used clinical treatment of KOA, mainly used in the treatment of early and middle KOA, has the advantages of simple operation, few side effects, and positive short-term and long-term effects [19]. In our earlier research, it has been suggested that warm acupuncture therapy could ameliorate injury in KOA rabbits [20]. In this experiment, “Heding, inner, and external knee eye” were selected as the treatment points. The needle body can enter the articular cavity through the inner and outer knee eye points. On the one hand, it can smooth the joints by the acupuncture effect of warm acupuncture; on the other hand, it can warm the meridians and dredge collaterals by the moxibustion effect of warm acupuncture. Related research suggests that the inner and outer knees are covered with abundant arteriovenous networks [21]. Stimulating the acupoint through the dual effects of warm needling and moxibustion can promote local blood circulation, reduce inflammatory substances and related metabolites, and provide a good internal environment for BMSCs transplantation.

On the basis of the above, it can be hypothesized that warm acupuncture combined with BMSCs might exert protective properties on KOA. To verify this hypothesis, this experiment investigates the potential protective effect of warm acupuncture combined with BMSCs transplantation on KOA cartilage tissue and proposes that this protective effect may explore new clinical treatment methods for KOA by inhibiting cell apoptosis.

## 2. Materials and Methods

### 2.1. Animals

A selection of 50 six-month-old New Zealand rabbits (female and male rabbits in each half) weighing 2.5–3 kg was provided from the Experimental Animal Center of Ningxia Medical University (certificate number: SCXK Ningxia 2015-0001). The animals were maintained under effectively fixed conditions of temperature (22–24°C), humidity (40–70%), photoperiod (12 h/12 h light/dark cycle), and food and water available ad libitum. The experiment was carried out in accordance with the current guidelines for the care of laboratory animals in Ningxia Medical University. All researchers who performed animal testing were blind.

### 2.2. Reagents

Rabbit bone marrow mesenchymal stem cells (RBXMX-01001) were purchased from Saiye (Suzhou, China); fetal bovine serum (FBS) (10270-106) was purchased from Gibco (NY, USA); DMEM (SH30022.01B) was purchased from HyClone (USA); the TUNEL assay kit (11684795910) was purchased from Roche (Switzerland); 0.25% trypsin-EDTA (C0201), penicillin-streptomycin liquid (C0222), L-glutamine (C0235), and DAPI staining solution (C1006) were purchased from Beyotime Biotechnology (Shanghai, China); anti-Bax (ab270742), anti-Bcl-2 (ab250782), and anti-Caspase-3 (ab32351) antibodies were purchased from Abcam (Cambridge, UK); 10% goat serum (G9023) was purchased from Sigma-Aldrich (Shanghai, China); the hematoxylin-eosin staining kit (G1120), modified safranin O-fast green FCF cartilage stain kit (G1371), toluidine blue O cartilage stain solution (G2543), EDTA decalcifying solution (E1171), and paraformaldehyde 4% (E1110) were purchased from Solarbio Science & Technology Co., Ltd. (Beijing, China); RNAprep Pure Tissue Kit (DP431) was purchased from Tiangen Biotech (Beijing, China); and RT-Master Mix (639505) was purchased from Takara Bio (Beijing, China).

### 2.3. Experimental Protocol

**Figure 1:**
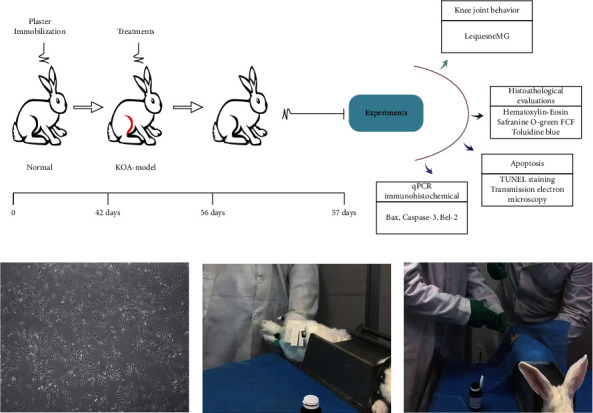
Experimental design. (a) The right hind limb of rabbits was positioned by plaster immobilization for 6 weeks. After the KOA model was built successfully, each intervention group was given different intervention in the same experimental environment, with 7 days as one course of treatment and the total of 2 courses. The next day after the treatment, knee joint structure and function were evaluated. (b) Photo of the 3^rd^-generation BMSCs under a light microscope. (c) Photo of warm acupuncture treatment. (d) Rabbits were injected with BMSCs in articular cavity.

The experimental protocol used is illustrated in Figure 1(a). New Zealand rabbits were randomly assigned to five groups, with each group comprising 10 rabbits: (a) blank group underwent no intervention and were fed normally; (b) KOA group underwent no intervention after the establishment of the KOA model; (c) in the warm acupuncture (WA) group, acupuncture was used at Dubi (ST 35), Neixiyan (EX-LE 4), and Heding (EX-LE 2) on the affected side, followed by using the warmth of moxa which is attached on the handle of the needles for warm acupuncture, and the procedure was carried out one time daily for 2 weeks; (d) in the BMSCs group, after routine sterilization with ethanol, 1 mL BMSCs were injected into the rat knee joint cavity of the right hind limb; each course of treatment was injected once; (e) in the warm acupuncture combined with BMSCs group, based on BMSCs treatment, warm acupuncture combined treatment was given, and, on the first day of each course, 1 mL BMSCs were injected into the rat knee joint cavity of the right hind limb, and warm acupuncture treatment was given for the remaining six days. The blank group, KOA group, and warm acupuncture group received NS under similar conditions to ensure the same conditions in each experimental group. That is, on the first day of each course, 1 ml normal saline was injected into the rat knee joint cavity of the right hind limb. The actual picture of the operation is shown in Figure 1(d).

### 2.4. Isolation and Culture of Rabbit BMSCs

Rabbit BMSCs were purchased from Cyagen Biotechnology Limited Company (Suzhou, China), and all procedures were approved by the Ethics Committee of the General Hospital of Ningxia Medical University (Yinchuan, China). Briefly, stem cells are isolated from the internal thread cryogenic vials and cultured in Dulbecco's modified Eagle's medium (DMEM), and 10% fetal bovine serum, 1 × insulin-transferrin-selenium, 2 mm L-glutamine, 100 U/mL penicillin, and 100 *μ*g/mL streptomycin were added. BMSCs were stored in a 37°C saturated humidity 5% CO_2_ incubator. BMSCs were identified and cultured to maturity, and all available bone marrow mesenchymal stem cells were collected for injection therapy [22]; the results of the culture performed are given in Figure 1(b).

Normal saline (NS), trypsin, and complete medium were preheated to 37°C during the preliminary experiment. The third-generation BMSCs cultured in vitro were digested with trypsin and observed under an inverted microscope. When the cells retracted to a round shape, the wall of the culture bottle was tapped to make the cells fall off. The cell suspension was collected, followed by centrifugation at 1000 r/min for 3 min, and the supernatant was suspended with NS. After adjusting the cell concentration to 1 × 10^6^/mL, the cell suspension was separated into a 1 mL syringe for reserve [23, 24].

### 2.5. KOA Model

The rabbit model of cartilage degeneration in KOA was reproduced by plaster immobilization in the right hind limb extension position for 6 weeks [25, 26], and the operation is performed as in Figure 1(c). The rabbit was simply placed in a supine position on the immobilizer, exposing the right knee joint. The knee joint was shaved, disinfected, and aseptically draped. Then the plaster bandage was folded after skin preparation for 8 layers, about 15 cm long, soaked in water, and then applied to the right knee of the rabbit. The lower end of the plaster was stuck at the ankle and the upper end exceeded 5 cm of the knee joint to straighten and then the knee joint was bandaged. The blood supply, swelling, and skin temperature of the right hind limb were observed daily after the establishment of the model. To verify the KOA model, 2 rabbits were randomly selected from the normal control group and the KOA group at 6 weeks after the operation, and the right knee joint was dissected by X-ray examination. Simultaneously, the general situation of mental state and bodyweight of the rabbits were evaluated. General situation assessment was combined with X-ray results to determine whether the replication of the KOA medium-term model was successful.

### 2.6. Modified Lequesne MG Score

Rabbits in each group were assessed by the Lequesne MG scale scoring system on the second day after successful modeling and the first day after the end of treatment to determine the changes in knee joint status and behavior [27]. The scoring system is as follows:

  (I) Local pain stimulus response (0 = no abnormal pain response; 1 = contraction of the affected limbs; 2 = contraction spasm of affected limbs with mild systemic reactions, e.g., tremor, back sucking, etc.; 3 = severe contraction spasm of affected limbs, tremor, chaos, and struggle)
  (II) Gait change (0 = no limp, normal running, and strong pedaling; 1 = limp slightly when running and strong foot pedal; 2 = obvious limp; 3 = the limbs not able to participate in walking and not able to touch the ground or pedal)
  (III) The range of motion of the joint (0 = above 90 degrees; 1 = 45 degrees to 90 degrees; 2 = 15 degrees to 45 degrees; 3 = 15 degrees)
  (IV) Degree of joint swelling (0 = no swelling, with bone markers clearly visible; 1 = mild swelling, with shallow bone mark; 2 = obvious swelling, with no bone markers)

### 2.7. Decalcification and Histopathological Analysis

The cartilage was fixed with 4% ice-cold paraformaldehyde for 2 days and then immersed in EDTA decalcification solution for 4 weeks after removal. The cartilage pieces were dehydrated and embedded in paraffin, successive 5 *μ*m sections made with a microtome (Leica, Germany). The sections were stained with hematoxylin and eosin (HE) staining, safranin O green FCF staining, and toluidine blue staining, and transmission electron microscopy (TEM) analysis was carried out.

### 2.8. Hematoxylin and Eosin (HE) Staining

The paraffin slices were dried at 70°C for 2 hours, deparaffinized, rehydrated, and stained with hematoxylin and eosin (HE), the histopathological changes of cartilage tissue were observed visually under a light microscope (Olympus BX-50, Tokyo, Japan) (×400), and then photographs were acquired. Injuries to the articular cartilage were assessed by the Mankin score [28]. The Mankin scoring criteria were as follows: (I) cartilage structure (0 = normal; 1 = irregular surface; 2 = rough surface of blood vessels; 3 = fissures entering the transitional layer; 4 = fissures entering the radiation layer; 5 = fissures entering the calcified layer; 6 = fissures penetrating the whole cartilage layer); (II) chondrocyte (0 = normal; 1 = diffuse increase; 2 = local increase; 3 = overall decrease); (III) cartilage matrix staining (0 = normal; 1 = shallow staining; 2 = shallow and uneven staining; 3 = basic nonstaining; 4 = decontamination); (IV) tidal line integrity (0 = integrity; 1 = damaged by blood vessels).

### 2.9. Safranin O Green Staining

The paraffin slices were dried at 70°C for 2 hours, deparaffinized, and rehydrated. Then slices were stained with fresh Weigert dye solution, differentiated 15 seconds with acid ethanol solution, and dyed with solid-green dye solution. The slices were washed with distilled water and soaked with safranin O dye solution. After washing with an acetic acid solution, tissue samples were deparaffinized and rehydrated by using xylene substitute and graded alcohols [29]. The injured sites were visually observed through a light microscope (Olympus BX-50, Tokyo, Japan) (×100) and photographs were acquired.

### 2.10. Toluidine Blue Staining

The paraffin slices were dried at 70°C for 2 hours, deparaffinized, and rehydrated by using a xylene substitute and graded alcohols. For toluidine blue staining, samples were immersed in toluidine blue O stain for 30 minutes and then washed thrice with distilled water for 2 minutes. After differentiating in acetone, the injured sites were visually observed through a light microscope (Olympus BX-50, Tokyo, Japan) (×100) and images were acquired [30].

### 2.11. Measurement of Transmission Electron Microscopy (TEM)

The cartilage blocks (5 mm × 5 mm × 2 mm) in the right knee were collected and fixed with 2% glutaraldehyde for 2 h at 4°C and washed thrice with 0.1 M phosphate buffer every 2 hours. The samples were then postfixed with 1% cold osmium tetroxide for 2 hours and rinsed twice with 0.1 M cacodylate buffer every 15 min. Then, they were dehydrated and embedded in epoxy resin. 0.5 *μ*m semithin sections were stained with 0.5% toluidine blue. After toluidine blue staining to orientate the injury site, fragments were cut by an ultramicrotome into 75 nm thick slices. The ultrathin slices were obtained on colloid-coated copper grids and double-stained with 0.4% uranyl acetate and 2% lead acetate [31]. Subsequently, morphological changes were observed and photographed by H-7650 electron microscope (Hitachi, Tokyo, Japan) (×3000 and ×20,000).

### 2.12. TUNEL Staining

Chondrocytic apoptosis was detected with the terminal deoxynucleotidyl transferase- (TdT-) mediated dUTP nick end labeling (TUNEL) using a cell death detection kit (Roche, Germany). In short, dewaxing was performed to slices in addition to a rehydration process followed by permeation with proteinase K for 15 minutes. Then, the DNA fragmentation of apoptotic chondrocyte bound the terminal deoxynucleotidyl transferase (TdT) enzyme in a reaction buffer. Slides were mounted with DAPI (Sigma, USA) for nuclear staining after rinsing with PBS for 10 minutes at room temperature [32]. In 6 different fields, the number of TUNEL-positive chondrocytes was counted and they were photographed blindly for each section using a laser scanning confocal microscope (Olympus FV1000, Japan). The results are expressed by the percentage of TUNEL-positive cells. The level of chondrocyte apoptosis is expressed as the ratio of apoptotic chondrocytes to total chondrocytes in each sample [33].

### 2.13. Immunohistochemistry Analysis

The fresh paraffin slices were dried at 70°C for 2 hours, deparaffinized, and rehydrated by using xylene substitute and graded alcohols. Then slices were placed in 3% hydrogen peroxide solution for 10 minutes. After rinsing with PBS three times, 10 mol/L urea was added to the slices at 37°C for 30 minutes. After irrelevant antigens were blocked using 10% goat serum for 30 minutes at room temperature, the primary antibodies (Bax, 1 : 200, ab32503; Bcl-2, 1 : 200, ab182858; Caspase-3, 1 : 200, ab32351; Abcam Group, USA) were incubated at 4°C overnight. The surface of cartilage slices was evenly covered with DAB, stained with hematoxylin, and soaked in 1% hydrochloric acid alcohol after rinsing with distilled water for 5 min. Tissue samples were deparaffinized and rehydrated by using xylene substitute and graded alcohols [34]. The images were observed using a microscope (Olympus BX-50, Tokyo, Japan) with high magnification (×200). Finally, the average optical density at a magnification of 200x was analyzed by Image-Pro Plus 6.0 software.

### 2.14. Evaluation of KOA by Quantitative Real-Time Polymerase Chain Reaction (qPCR)

KOA-associated gene expression was measured using qPCR. The cartilage was homogenized in RNAprep Pure Tissue Kit (Tiangen Biotech, Beijing, China), and total RNA was extracted according to the manufacturer's protocol. Then, 500 ng of total RNA was subjected to reverse transcription using RT-Master Mix (Takara Bio, Beijing, China) according to the manufacturer's protocol to generate complementary DNA (cDNA). The cDNA was serially diluted 10-fold with sterilized distilled water and qPCR was performed to detect the expression of cartilage-associated marker genes. The qPCR reaction mixture (10 *μ*L) was prepared using a KAPA SYBR Fast qPCR Kit (Nippon Genetics, Tokyo, Japan) according to the manufacturer's protocol. Specific primers were used at final concentrations of 100 nM. Amplification was performed using a QuantStudio 5 Real-Time PCR Systems thermal cycler (Thermo Fisher Scientific, Waltham, MA, USA). The cycling profile included one cycle of 95°C for 5 min, followed by 40 cycles of 95°C for 3 s and 60°C for 30 s, with a final stage of melting curve analysis. GAPDH RNA expression was also measured and used as the internal sample control. The value of each gene expression was calculated using the 2^∆∆^Ct method and normalized to GAPDH levels [35–37]. Primer sequences and target gene names are as follows: Bax (forward 5′-CGA GTC CAC CAA GAA GCT GA-3′/reverse 5′- GGC AGC GAT CAT CCT CTG TA-3′), Bcl-2 (forward 5′-TCC TTC CAG CCT GAG AGC AAC C-3′/reverse 5′-TGG ACC ACA GGT GGC ACA GG-3′), Caspase-3 (forward 5′-CTA AGC CAC GGT GAT GAA GGA GTC-3′/reverse 5′-CAC TGT CTG TCT CGA TGC CAC TG-3′), and GAPDH (forward 5′-GGC ATG GAG TCG TGT GGC ATC-3′/reverse 5′-CGT GTT GGC GTA CAG GTC CTT G-3′).

### 2.15. Data Analysis

Data were expressed as mean ± SEM, and the statistical analysis of the results was evaluated by one-way ANOVA followed by Dunnett's test. *p* < 0.05 was considered statistically significant.

## 3. Results

### 3.1. The Rabbit KOA Model Was Established Successfully

**Figure 2:**
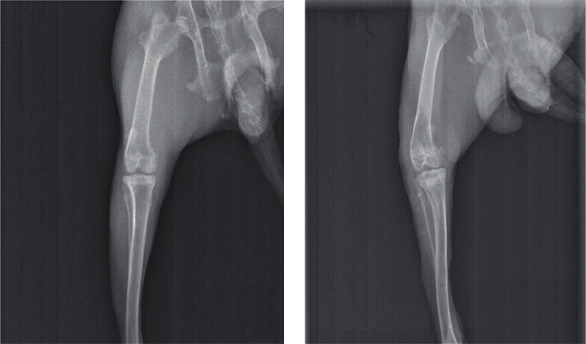
The rabbit KOA model was established successfully by X-ray. (a) Blank group; (b) model group.

**Figure 3:**
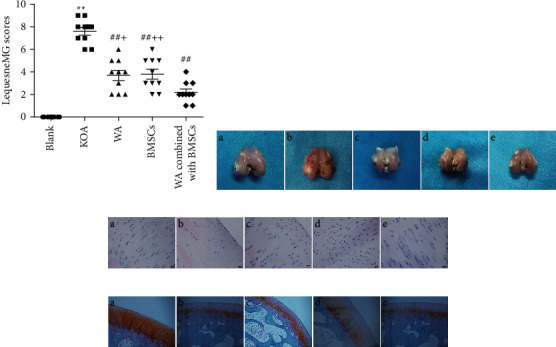
Effect of warm acupuncture combined with BMSCs on behavior and histomorphology alterations in KOA rabbit. (a) Lequesne MG scores in KOA rabbit. Data are expressed as mean ± SEM (*n* = 10). ^*∗∗*^*p* < 0.01 versus blank group, ^##^*p* < 0.01 versus KOA group, and ^+^*p* < 0.05 and ^++^*p* < 0.01 versus WA combined with BMSCs group. (b) Example images of the rabbit cartilage tissue of knee joint. A: blank group; B: KOA group; C: warm acupuncture (WA) group; D: BMSCs group; E: WA combined with BMSCs group. (c, d) Representative photomicrographs of HE (×400) and safranin O green staining (×100) of the rabbit cartilage tissue of knee joint. A: blank group; B: KOA group; C: warm acupuncture group; D: BMSCs group; E: warm acupuncture combined with BMSCs group.

Compared with the blank group, KOA rabbits generally had dim hair color, poor spirit, less diet, and poor endurance, and the right knee joint had more fluid and showed joint swelling, refused to press, and had claw scratches. The X-ray results show that the gap of the knee joint of the KOA rabbits was narrowed, the bone density was increased, the intercondylar crest was hyperplasia, the femur was bent, and the patella was turned over malformation (Figure 2(b)). The characteristics are consistent with KOA visually, some of the articular cartilage is missing, the surface is incomplete, and the synovial tissue is congested and hyperemia (Figure 3(b)).

### 3.2. Warm Acupuncture Combined with BMSCs Improved Knee Joint Behavior Deficiency Caused by KOA

KOA rabbits showed a higher behavior score than the blank group (Figure 3(a), *p* < 0.01). However, the increase in the Lequesne MG score was reduced significantly (Figure 3(a), *p* < 0.01) in the warm acupuncture combined with the BMSCs group compared to that in the KOA group. In addition, the effect of the warm acupuncture combined with BMSCs is better than that of warm acupuncture group and BMSCs group separately (Figure 3(a), *p* < 0.05 and *p* < 0.01).

### 3.3. Warm Acupuncture Combined with BMSCs Delayed the Histopathological Damage Induced by KOA

#### 3.3.1. H&E Staining

In the blank group, articular cartilage structure was good, the cells were uniformly distributed, the cells were evenly distributed, there was single column arrangement, and extracellular matrix staining was uniform. There were cracks in the rough surface of cartilage, the number of cartilage cells was obviously reduced, the distribution was sparse, shallow cells experienced pressured deformation, the hierarchical structure was disturbed, and the cracks were visible locally through the radiation layer. However, the cartilage structure was normal, without cracks, the tidal line structure was basically intact, the chondrocytes were arranged in a relatively regular manner, there was no clustering phenomenon, and the cartilage matrix staining was normal in the warm acupuncture combined with BMSCs group (Figure 3(c)). Therefore, compared with the blank group, the Mankin score of the KOA group was significantly increased (*p* < 0.05); Compared with the KOA group, the Mankin scores of the three intervention groups were significantly lower (*p* < 0.05), and the Mankin score of the combined treatment group was significantly lower than that of the treatment group separately (Table 1, *p* < 0.05).

#### 3.3.2. Safranin O-Green Staining

Chondroitin sulfate and keratin sulfate in the cartilage matrix were acidic and red after reacting with slightly alkaline safflower O. Collagen fibers in the superficial layer of cartilage and subchondral bone contain a large number of alkaline amino acids, which are green when combined with acid fixation. In the blank group, the cartilage structure was intact, the layers were clear, the cells were arranged in a single row, the cartilage matrix was red, and the subchondral bone was green (Figure 3(d)-A). In the KOA group, the superficial layer was rough, the chondrocytes were arranged in disorder, the number of cells was reduced, longitudinal cracks were formed in the superficial layer of cartilage, the severity of lamina O staining was weakened, 2/3 of the upper cartilage lost staining, and the nonuniform staining of subchondral bone was observed in the solid green staining (Figure 3(d)-B). Warm acupuncture combined with BMSCs has a complete cartilage structure, clear layers, and uniform saffron-solid green color (Figure 3(d)-E).

#### 3.3.3. Toluidine Blue Staining

**Figure 4:**
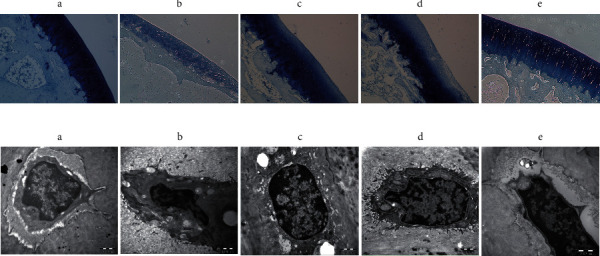
Histological evaluation. (A) representative photomicrographs of toluidine blue staining (×100) of the rabbit cartilage tissue of knee joint. (a) Blank group; (b) KOA group; (c) warm acupuncture (WA) group; (d) BMSCs group; (e) warm acupuncture combined with BMSCs group. (B) Effects of warm acupuncture combined with BMSCs on ultrastructural alterations in cartilage cells of knee joint (20000x magnification; bar = 1000 nm).

The cartilage tissue in the blank group was clearly arranged, the structure was intact, the number of chondrocytes was normal, and the toluidine blue staining was uniform (Figure 4(a)). Meanwhile, the cartilage tissue of the KOA group was severely damaged, involving the entire cartilage layer, with reduced chondrocytes and weak positive toluidine blue staining (Figure 4(a)). Also, the number of chondrocytes in the warm acupuncture combined with the BMSCs group increased, presenting a single columnar neat arrangement, and the deep staining of the cartilage was not uniform (Figure 4(a)). Interestingly, under the electron microscope, the above-mentioned histological changes showed highly consistent changes (Figure 4(b)).

### 3.4. Effects of Warm Acupuncture Combined with BMSCs on Apoptosis in KOA Rabbits

#### 3.4.1. Warm Acupuncture Combined with BMSCs Reduced the Formation of Apoptotic Body

**Figure 5:**
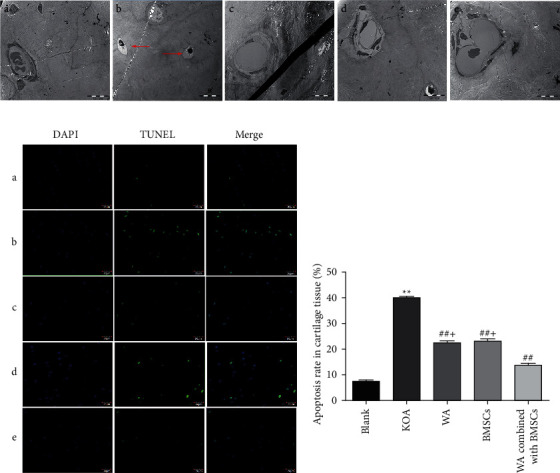
Effect of warm acupuncture combined with BMSCs on rabbit cartilage tissue of knee joint after KOA damage. (a) Representative images of apoptosis in vascular knee joint cartilage (×3000, bar = 10 *μ*m). A: blank group; B: KOA group; C: warm acupuncture (WA) group; D: BMSCs group; E: WA combined with BMSCs group. The red arrows represent apoptotic body. (b) Representative fluorescence photo of TUNEL staining in cartilage tissue of knee joint. A: blank group; B: KOA group; C: warm acupuncture (WA) group; D: BMSCs group; E: WA combined with BMSCs group. (c) Quantitative analysis of the TUNEL-positive cells. Data are expressed as mean ± SEM (*n* = 10). ^*∗∗*^*p* < 0.01 versus the blank group, ^##^*p* < 0.01 versus the KOA group, and ^+^*p* < 0.01 versus the warm acupuncture combined with the BMSCs group.

Transmission electron microscopy was used to observe the changes of apoptotic body. As demonstrated in Figure 5(a), chondrocytes were normally oval in shape and were distributed in the cartilage matrix, with full nuclei; chromatin was evenly distributed; and rough endoplasmic reticulum, Golgi body, and mitochondria scattered in the cytoplasm. Regular collagen fibers could be seen outside the cells, blood vessels were full, and red blood cells were arranged neatly. However, the apoptotic body was observed in KOA chondrocytes but not in the warm acupuncture combined with BMSCs group. Likewise, no apoptotic body was observed in the blank group or in the warm acupuncture group and BMSCs group.

In the same way, the results of TUNEL staining are similar, as shown in Figure 5(b). A large number of TUNEL-positive cells were densely observed in KOA rabbits, while this was rarely observed in the blank group. In comparison with the KOA group, warm acupuncture combined with BMSCs substantially reduced the TUNEL-positive cells (Figure 5(c), *p* < 0.01).

#### 3.4.2. Warm Acupuncture Combined with BMSCs Regulated the Expression of Apoptosis-Related Proteins

**Figure 6:**
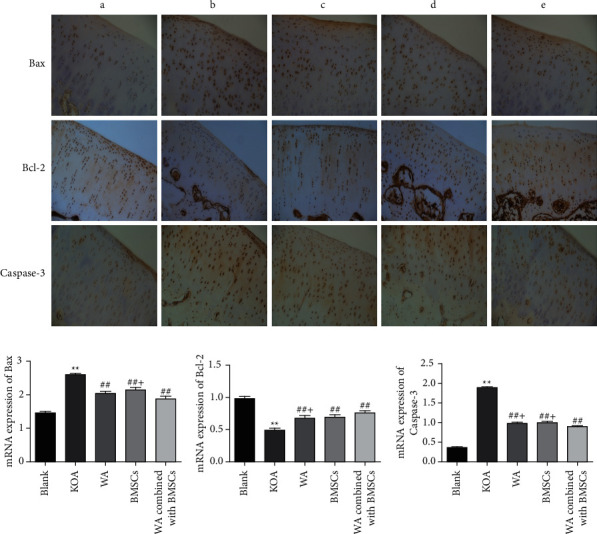
Effects of warm acupuncture combined with BMSCs on the expressions of Bax, Bcl-2, and Caspase-3 in cartilage tissue of knee joint. (a) Immunohistochemical results of Bax, Bcl-2, and Caspase-3 for the different groups. (b–d) Quantification of qPCR data of Bax, Bcl-2, and Caspase-3 in cartilage tissue of knee joint. Data are expressed as mean ± SEM (*n* = 10). ^*∗∗*^*p* < 0.01 versus the blank group, ^##^*p* < 0.01 versus the KOA group, and ^+^*p* < 0.05 versus the warm acupuncture combined with the BMSCs group.

The immunohistochemical analysis result indicated that the KOA group exhibited upregulated expression of Bax and Caspase-3 and downregulated expression of Bcl-2 compared with the blank group. The warm acupuncture combined with BMSCs can significantly inhibit the increase in the expression of Bax and Caspase-3 and reduce the expression of Bcl-2 (Figure 6(a)). At the same time, real-time RT-PCR analysis indicated that, compared with the KOA group, warm acupuncture combined with BMSCs can reduce mRNA expression of Bax and Caspase-3 (*p* < 0.01 and *p* < 0.01; Figures 6(b) and 6(d)). The results also indicated that, compared with the blank group, the expression of Bcl-2 in the KOA group was downregulated, while the mRNA level of Bcl-2 in the warm acupuncture combined with BMSCs group was significantly increased (*p* < 0.01, Figure 6(c)).

## 4. Discussion

The main finding of the present study is that warm acupuncture combined with BMSCs can markedly improve the impairments of knee joint behavior and decrease the extent of cartilage tissue pathology relative to that of the rabbits subjected to KOA. Moreover, our results exhibited that warm acupuncture combined with BMSCs intervention can decrease the formation of apoptosis body and reversed the expression of apoptosis-related proteins Bax, Bcl-2, and Caspase-3 induced by KOA. These results suggest that warm acupuncture combined with BMSCs offers potential protective effects against KOA rabbits, and their protective effects may be related to the inhibiting chondrocyte apoptosis.

The Lequesne MG index reflects the severity of KOA [38]. The results showed that, before treatment, the four groups of KOA model rabbits had obvious knee joint pain stimulation. The knee joint was swollen and the range of motion was significantly reduced, which was very similar to the clinical symptoms of KOA, indicating that the model was successfully replicated. After treatment, the Lequesne MG score of the warm acupuncture combined with BMSCs was significantly lower than that of the model group, the degree of knee swelling reduced, the range of motion increased, and the knee joint compression pain response was weakened. The improvement of the Lequesne MG index in the combination group was better than that of the single-treatment group, suggesting that the combination group of warm acupuncture and BMSCs can effectively restore joint function, and the effect is better than that of the single-treatment group. Similarly, the gross observation shows similar results. In the warm acupuncture combined with BMSCs group, the articular cartilage injury was mild, the amount of synovial fluid was yellowish, sticky, and slippery, and there was no significant increase, suggesting that the treatment could improve the KOA articular surface injury. In addition, morphological staining can further verify the therapeutic effect of warm acupuncture combined with BMSCs on KOA.

Some evidence suggested that apoptosis plays a vital role in the pathogenesis of KOA [39, 40]. The degenerative changes of articular cartilage are the main pathological features of KOA. But, at this time, the morphological changes of cartilage cells are reversible, which may be restored by activating survival signal or active death by activating apoptotic signal [41, 42]. If the corresponding interventions are not given in time, a large number of cartilage cells will undergo apoptosis, which will aggravate cartilage tissue damage and eventually lose joint function. Therefore, it is feasible and reasonable to protect against knee cartilage by inhibiting apoptosis. In this study, TEM showed that the formation of apoptotic body and cartilage injury substantially increased after KOA; nevertheless, the strengthening of these effects was mitigated by warm acupuncture combined with BMSCs after the intervention. Activated Caspase-3 can directly initiate the apoptotic process and occupy the central position in the process of apoptotic signal transduction; Bax is one of the apoptotic proteins, which can activate Caspase-3 protease and initiate the apoptotic process; Bcl-2 is a classic apoptotic antagonist protein, which can block the channels formed by Bax. In consistency with a previous study, our results showed that the protein levels of Bax and Caspase-3 increased, and the protein levels of Bcl-2 decreased. Warm acupuncture combined with BMSCs after treatment inhibited apoptosis by reversing these changes after KOA.

Some studies have shown that the level of Bcl-2 decreased and those Bax and Caspase-3 increased after KOA; that is, apoptosis was activated [8, 43–45]. Likewise, the results of the present study revealed that KOA induced apoptosis and degenerative changes in the rabbit knee cartilage, and the intervention of warm acupuncture combined with BMSCs can reverse these changes. That is to say, the combination therapy inhibits apoptosis activation by increasing the expression of Bcl-2 and decreasing the expressions of Bax and Caspase-3. These results suggested that the cartilage protection activities of warm acupuncture combined with BMSCs on KOA may be through inhibiting apoptosis.

In conclusion, this study is the first to report that warm acupuncture combined with BMSCs transplantation can effectively inhibit KOA cartilage tissue injury in rabbits. The effect is obviously better than that of simple warm acupuncture and simple BMSCs, confirming the synergistic effect. Also, warm acupuncture combined with BMSCs transplantation may reduce injury to KOA rabbit cartilage tissue by reducing the expressions of Bax and Caspase-3, raising Bcl-2 levels, and inhibiting cartilage apoptosis. Therefore, it can be speculated that the warm acupuncture combined with BMSCs may inhibit chondrocyte apoptosis on KOA rabbits, delaying KOA cartilage tissue injury, which promoted chondrocytes survival. However, the experiments in this study have several limitations, particularly on the mechanism of warm acupuncture combined with BMSCs to treat KOA, as well as lack of studies exploring the primary mechanism of protection. More cartilage effects and various relevant cell survival mechanisms of warm acupuncture combined with BMSCs on KOA need to be further explored. At present, the targeted differentiation mechanism and influencing factors of BMSCs are not clear. Further studies are needed to determine how to improve the directed differentiation rate of BMSCs. In addition, the current experiment is still some initial animal experiment, and advanced animal and clinical studies are needed. Long-term large-scale randomized clinical trials are needed to improve the interpretation, so as to determine the level of efficacy in future studies. Although there are still many questions about the application of BMSCs which need to be further studied, BMSCs should not be ignored as the direction of future cartilage repair. It is believed that, with the deepening of the research and the promotion of the application, the tissue-engineered cartilage will be better applied in clinic.

## Figures and Tables

**Table 1:** Mankin scores (*n* = 10).

Group	Mankin scores
Control group	1.0 ± 0.67
KOA group	8.3 ± 1.34^*∗∗*^
Warm acupuncture group	4.1 ± 1.20^##+^
BMSCs group	4.4 ± 1.17^##+^
Warm acupuncture combined with BMSCs group	3.0 ± 0.94^##^

Data are expressed as mean ± SEM (*n* = 10). ^*∗∗*^*p* < 0.01 versus the blank group; ^##^*p* < 0.01 versus the KOA group; ^+^*p* < 0.01 versus the warm acupuncture combined with BMSCs group.

## Data Availability

The data used to support the findings of this study are available from the corresponding author upon request.
